# Aerobic Exercise under Cold Conditions May Affect the Cardiac Structure by Elevating the Galectin 3 and CK-MB Levels

**DOI:** 10.5114/jhk/213943

**Published:** 2026-04-02

**Authors:** Saime Ozbek Sebin, Engin Sebin, Basak Gulakar, Cebrail Gencoglu, Serhat Ozbay, Suleyman Ulupinar, Konca Altinkaynak, Abdullah Bora Ozkara

**Affiliations:** 1 Department of Physiology, Medical Faculty, Atatürk University, Erzurum, Turkey.; 2 Department of Medical Biochemistry, Erzurum City Hospital, Erzurum, Turkey.; 3 Department of Physiology, Medical Faculty, Kafkas University, Kars, Turkey.; 4 Department of Physical Education and Sport, Faculty of Sport Sciences, Erzurum Technical University, Erzurum, Turkey.; 5 Department of Medical Biochemistry, Medical Faculty, Health Sciences University, Istanbul, Turkey.; 6 Department of Physical Education and Sports, Karadeniz Technical University, Trabzon, Turkey.

**Keywords:** aerobic exercise, Galectin 3, CK-MB, cold weather, cardiac injury

## Abstract

This study aimed to evaluate the myocardial effects of aerobic exercise in cold weather in healthy individuals by measuring galectin-3 (Gal-3) and CK-MB levels. Forty-one individuals (12 professional athletes [age: 21.91 ± 2.60 years, body mass: 63.5 ± 8.4 kg, BMI: 21.7 ± 1.6 kg/m^2^], 14 physically active [age: 21.95 ± 2.30 years, body mass: 65.5 ± 10.3 kg, BMI: 22.4 ± 1.4 kg/m^2^], and 15 sedentary individuals [age: 21.40 ± 2.64 years, body mass: 62.5 ± 7.4 kg, BMI: 22.2 ± 1.7 kg/m^2^]) participated in this study. Athletes and physically active individuals performed 40 minutes of acute aerobic running exercises at 0°C and 20°C environmental temperatures. Gal-3 and CK-MB levels were measured pre- and post-exercise. Baseline Gal-3 levels of professional athletes (p < 0.001) and physically active individuals (p = 0.048) were significantly higher than those of sedentary individuals. Two-way ANOVA showed significant interaction (p = 0.035) between temperature (0°C vs. 20°C) and exercise (pre- vs. post-exercise). At 0°C, there was a significant increase in post-exercise serum Gal-3 levels when compared with pre-exercise values (p < 0.001, +7.1%), whereas no significant difference was observed at 20°C. Moreover, no significant interaction was found for CK-MB levels (p = 0.306) between the temperature (0°C vs. 20°C) and exercise (pre- vs. post-exercise). Additionally, there was a significant difference between pre- and post-exercise values at the 0°C environmental temperature for serum CK-MB levels (p < 0.001), while there was no significant difference at 20°C. This study demonstrates that regular exercise is associated with biochemical abnormalities that may affect cardiac structure and function. Aerobic exercise under cold conditions may cause myocardial injury.

## Introduction

Cardiovascular disease (CVD) is an important global problem with an increasing prevalence, high mortality, and morbidity. Regular exercise is effective in the treatment and rehabilitation of CVD, such as coronary artery disease (CAD), and prevention of the development of fatal cardiac injuries such as myocardial infarction (Carvalheira-dos-Santos et al., 2019). However, intense exercise, especially long-term strenuous endurance exercise, may cause acute and chronic effects such as myocardial fibrosis, right ventricular structural changes, and arrhythmias (Wilson et al., 2011). One hour of cycling exercise at 50% of maximum workloads has been shown to increase lipid peroxidation, oxidative stress, and antioxidant capacity (Williamson-Reisdorph et al., 2023).

Galectin-3 (Gal-3), a member of the lectin family, is upregulated in the myocardium following heart failure, correlating with infarct size and serving as a biomarker for cardiac fibrosis and mortality risk (Cowling et al., 2019; Gagno et al., 2019). It is expressed in various tissues, including the heart, brain, liver, and immune cells (Hättasch et al., 2014). Gal-3 promotes inflammation by inducing cytokines and contributes to lipid endocytosis, apoptosis, and oxidative stress. It is also implicated in atherosclerosis through activation of proinflammatory pathways affecting endothelial cells, smooth muscle cells, and macrophages (Gao et al., 2020; Papaspyridonos et al., 2008).

Various studies investigating the change of Gal-3 serum levels due to exercise found that post-exercise Gal-3 levels were higher than the baseline. In a study with trained athletes who ran a 60-km ultramarathon, Gal-3 levels were higher than the baseline levels. Endurance exercise may cause adverse effects on the cardiac structure (Salvagno et al., 2014). Another study with non-elite marathon runners who ran 30 km (Hättasch et al., 2014) found that post-exercise Gal-3 levels were higher than pre-exercise levels, while the ventricular function did not change in cardiac magnetic resonance imaging. Le Goff et al. (2020) showed that Gal-3 levels increased post-exercise.

Three cytoplasmic isozymes of creatine kinase (CK) may be found in three different types of tissues: CK-MB in the heart, CK-MM in muscles, and CK-BB in the brain. As a biomarker for coronary syndromes, including acute myocardial infarction (AMI), CK-MB is crucial in clinical medicine. Furthermore, it performs better in diagnosing reinfarction than cardiac troponins. In clinical medicine, CK-MB is utilized as a possible supplementary test for the diagnosis of cardiovascular disorders, in conjunction with other biomarkers such as cardiac troponins. Levels of total CK and CK-MB are significant prognostic indicators that are connected with infarct size (Aydin et al., 2019; Xu et al., 2020).

Exercise and cold exposure are known metabolic stimulants that help prevent obesity-related complications (Ozbay et al., 2020; Ulupinar et al., 2021). However, cold-weather exercise increases sympathetic activity, blood pressure, peripheral resistance, and myocardial oxygen demand, potentially raising cardiovascular risk through thrombogenicity, bronchoconstriction, proinflammatory cytokine release, and arrhythmias (Cappaert et al., 2008; Nagelkirk et al., 2012; Park et al., 2020). Parasympathetic disruption following exercise has been found to improve parasympathetic reactivity and modulate heart rate variability with cold water immersion (Ravier et al., 2022). While regular aerobic exercise improves endothelial function, cold exposure may impair flow-mediated dilation (Valtonen et al., 2020). Five days of partial body cryotherapy after exercise has been shown to enhance athletic performance (Kim et al., 2025). A study by Sebin et al. (2024) found elevated C1q/TNF-related protein 3 (CTRP3) levels after aerobic exercise under cold conditions, highlighting temperature-dependent physiological effects. Notably, no studies have assessed the cardiac impact of cold-weather aerobic exercise using Gal-3 and CK-MB levels.

Gal-3 and CK-MB reflect different but complementary aspects of heart stress. Gal-3 is linked to inflammation and early cardiac remodeling and is recognized for its prognostic role in heart failure. It responds to physical stress and can indicate tissue-level changes even without visible heart damage. CK-MB, on the other hand, is a classic marker of myocardial cell stress and is more specific to the heart than total CK. While Gal-3 captures longer-term inflammatory changes, CK-MB helps detect short-term myocardial strain. Using both markers offers a broader view of the heart’s response to exercise, especially under cold conditions.

Therefore, in this study, we aimed to evaluate the cardiovascular system effects of exercise in cold weather in healthy young athletes. We investigated the acute effects of aerobic exercise performed under different ambient temperature conditions—specifically 0°C and 20°C—on serum levels of Galectin-3 (Gal-3) and creatine kinase-MB (CK-MB) in healthy individuals with varying physical activity levels. By evaluating changes in these cardiac biomarkers before and after exercise in both cold and moderate environments, the study sought to determine whether cold exposure would enhance physiological cardiac stress responses and to explore Gal-3’s potential utility as an early indicator of exercise-induced myocardial strain.

## Methods

### Study Design and Participants

This study was approved by the ethics committee of the Erzurum Regional Training and Research Hospital, Erzurum, Turkey (approval code: #ID 2021/03-54; approval date: 01 February 2021), and conducted following the principles of the Declaration of Helsinki. Informed consent was obtained from all participants. Although G*Power analysis indicated a minimum sample size of 41 participants, 45 individuals were initially recruited to account for potential dropouts. Participants were randomly assigned to start with either a cold or a moderate temperature session using a computerized list. The order was kept hidden in sealed envelopes until the sessions began. While blinding the participants and exercise supervisors was not possible due to perceptible temperature conditions, the laboratory team analyzing the blood samples and researchers handling the statistical analysis were unaware of the group assignment. This approach helped reduce potential bias in the outcome measurement.

Due to logistical constraints, four participants withdrew, resulting in a final sample of 41 (age: 21.5 ± 2.4 years; BMI: 22.1 ± 1.6 kg/m^2^; n = 12 professional athletes, n = 14 physically active individuals, n = 15 sedentary individuals) (a post-hoc power analysis confirmed that the final sample size was still sufficient to achieve the required statistical power). Participants were grouped by their training background: professional athletes (≥ 5 years of competitive experience), physically active (non-competitive exercise ≥ twice a week), and sedentary individuals. Only athletes and active individuals completed the exercise protocols. Each of them underwent 40 min of running at 0°C and 20°C in a randomized crossover design, with a 72-hour washout period between sessions. A 40-min aerobic exercise session at 70% of the heart rate reserve was chosen following well-validated exercise protocols known to stimulate both cardiovascular and metabolic systems without compromising participants’ safety (Bayles, 2023; Matsuo et al., 2014). This exercise duration was consistent with previous studies examining short-term changes in cardiac biomarkers under moderate-to-vigorous aerobic workloads and had been commonly utilized in sports science research (Eijsvogels et al., 2015; Le Goff et al., 2020; Miles and Schneider, 1993; Salvagno et al., 2014).

All blood samples were collected at 20°C. Cold exposure acclimatization was considered comparable across active groups due to the regional climate. Exclusion criteria included age outside 18–25, cardiovascular/chronic disease, medication or supplement use, smoking, or refusal to participate. Participants followed fasting and dietary guidelines; they also refrained from stimulants during the study. Individuals with sensitivity to cold, who were on a special diet program, were not included.

The following were the requirements for inclusion: those between the ages of 18 and 25 who did not have any long-term health issues that required frequent prescription medication.

All experimental sessions were conducted indoors in a climate-controlled sports laboratory at the Erzurum Technical University (Erzurum, Turkey) to standardize environmental exposure. Each participant completed two experimental sessions—one at 0°C and one at 20°C—scheduled between 15:30 and 16:30 h (local time, UTC +3) to minimise circadian variation in cardiovascular and hormonal responses (Atkinson and Reilly, 1996). Room humidity was maintained at 40 ± 3% (Model HMT337, Vaisala, Vantaa, Finland), and the temperature was verified at five floor-level points using calibrated digital thermometers (Testo 926, Testo SE, Lenzkirch, Germany).

### Pre-Exercise Controls

Participants arrived after a 3–4 h post-absorptive period (water ad libitum) and were instructed to:

- avoid caffeine and alcohol for 24 h,
- abstain from vigorous exercise, non-steroidal anti-inflammatory drugs, and any supplements for 48 h,
- maintain habitual dietary intake, but record the last meal for replication at the second visit. Compliance was confirmed via a standardised checklist on arrival.

At 20°C, participants wore light sportswear of their choice. At 0°C, they added a three-layer clothing system (base layer + fleece mid-layer + wind-proof shell) to comply with occupational cold-exposure guidelines (Oi, 2025).

### Exercise Protocol

After a 10-min dynamic warm-up, except for the sedentary participants, individuals ran for 40 min after reaching 70% of their maximum heart rate (Heart Rate Reserve Method (Karvonen Formula) (Archundia-Herrera et al., 2017).

The running intensity was calculated using the following formula: Target HR zone = [(HR_max_ − HR_rest_) * % intensity) + HR_rest_] (Batitucci et al., 2019; Karvonen, 1957). Testing was performed indoors to maintain consistent temperature conditions at 0°C and 20°C. Heart rate values were continuously monitored using telemetric heart rate monitors (S610i, Polar Electro Oy, Kempele, Finland) to ensure participants maintained the target intensity of 70% HRR (Bayles, 2023).

### Biochemical Measurements

10 cc of venous blood taken from all of the participants before (at 15.25) and after exercise (at 16.35) was centrifuged, and sera were separated. Samples were stored at −80°C until the day of the measurement. Gal-3 levels were evaluated using the Human galectin-3 ELISA platinum kit (Ebioscience, Austria) from samples thawed on the day of the experiment. The assay was performed according to the manufacturer’s recommendations and showed a limit of detection of 2.9 ng/mL, an intra- and an inter-assay coefficient of variation (CV) of 5.4% and 7.5%, respectively.

CK-MB levels in serum samples were studied using Siemens kits on a Siemens Atellica IM analyzer. Baseline and post-exercise samples were taken from the participants for Gal-3; however, baseline CK-MB levels were not included because baseline values are typically low in healthy individuals and are more relevant for detecting acute cardiac stress post-exercise.

### Statistical Analysis

All analyses were performed using IBM SPSS Statistics for Windows, Version 21.0 (IBM Corp., Armonk, NY, USA). The significance level for all statistical tests was determined using a two-tailed approach. Independent sample *t*-tests (between males vs. females) and one-way ANOVA were performed to compare the baseline Gal-3 and anthropometric characteristics of the sub-groups. Repeated measures two-way ANOVA was chosen to assess the time (pre- vs. post-exercise) and temperature interactions (0°C vs. 20°C) for all participants performing the exercise protocols. Repeated measures three-way ANOVA was used to determine the time (pre- vs. post-exercise) and environmental temperature (0°C vs. 20°C) interactions in the different sub-groups (males vs. females, professional athletes vs. physically active individuals).

Before conducting parametric tests, the assumptions of normality (Shapiro-Wilk test), homogeneity of variances (Levene’s test), and sphericity (Mauchly’s test) were examined and confirmed. Greenhouse-Geisser corrections were applied when sphericity was violated. Gal-3 and CK-MB were log-transformed to meet normality assumptions. Descriptive statistics were presented as mean ± SD or median [IQR] as appropriate.

Effect sizes were calculated to enhance the interpretation of statistical findings. Cohen's *d* values were computed for pairwise comparisons to indicate the magnitude of differences between pre- and post-exercise measurements (Cohen, 2013). Additionally, partial eta squared (ηp^2^) values were reported for ANOVA statistics to assess the proportion of variance explained by each factor. These effect sizes provide complementary information to *p*-values, highlighting the practical significance of observed differences.

## Results

Table 1 presents anthropometric characteristics and Gal-3 values obtained before the study commencement. According to the analysis, there was a significant difference in the Gal-3 levels. The Bonferroni post hoc test indicated that baseline Gal-3 levels of professional athletes (*p* < 0.001) and the physically active group (*p* = 0.048) were significantly higher than that of the sedentary group. Additionally, body height, body mass, and BMI values were significantly higher in males compared to females (*p* < 0.001 for all).

**Table 1:** Comparison of anthropometric characteristics of the participants, and Gal-3 values at the baseline according to gender and training status variables.

	Professional (n = 12)	Physically Active (n = 14)	Sedentary (n = 15)	F	*p*
Age (year)	21.91 ± 2.60	21.95 ± 2.30	21.40 ± 2.64	0.194	0.825
Body Height (cm)	170.6 ± 7.1	170.4 ± 9.0	167.6 ± 4.7	0.807	0.454
Body Mass (kg)	63.5 ± 8.4	65.5 ± 10.3	62.5 ± 7.4	0.435	0.650
BMI (kg/m^2^)	21.7 ± 1.6	22.4 ± 1.4	22.2 ± 1.7	0.594	0.557
Baseline Gal-3 (ng/mL)	21.7 ± 5.3	18.7 ± 2.8	15.2 ± 2.7	10.196	< 0.001
	Females	Males	t	*p*
Age (year)	21.8 ± 2.4	21.3 ± 2.5	0.676	0.503
Body Height (cm)	163.6 ± 4.7	174.0 ± 5.0	−6.667	< 0.001
Body Mass (kg)	55.6 ± 3.2	70.3 ± 5.4	−9.978	< 0.001
BMI (kg/m^2^)	20.7 ± 1.0	23.1 ± 1.1	−6.935	< 0.001
Baseline Gal-3 (ng/mL)	17.6 ± 5.2	18.9 ± 3.8	−0.851	0.400

Notes; BMI = Body mass index; values in bold indicate statistically significant differences (p < 0.05)

Two-way ANOVA showed a significant interaction (F = 4.705, *p* = 0.035, ηp^2^ = 0.086) between the temperature (0°C vs. 20°C) and exercise (pre- vs. post-exercise) for Gal-3 levels.

Additionally, there was a significant main effect of exercise (F = 13.147, *p* < 0.001, ηp^2^ = 0.208), indicating that post-exercise Gal-3 levels were significantly higher across all temperature conditions. There was a significant increase in post-exercise values of serum Gal-3 at the 0°C environmental temperature (*p* < 0.001, *d* = 0.33) compared with pre-exercise values, whereas no significant difference was found (*p* = 0.368, *d* = 0.09) at 20°C (Figure 2, left panel). Furthermore, paired Student *t*-test analysis showed that Gal-3 elevation from baseline at 0°C (t = 2.317, *p* = 0.029, *d* = 0.060) was significantly higher compared to 20°C (+7.1% and +2.1%, respectively).

**Figure 1:**
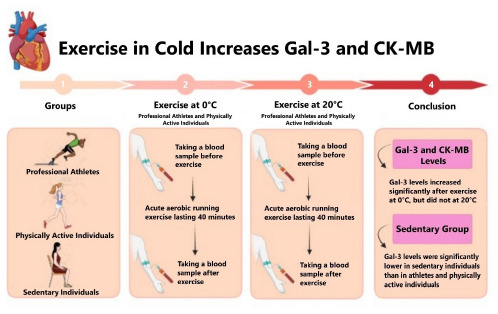
Graphical abstract.

**Figure 2:**
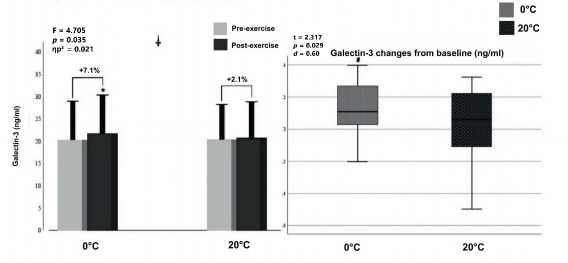
Graphical representation of Gal-3 concentration pre- and post-exercise (left panel) and net change from baseline (right panel). Notes: *: significantly different from pre-exercise value; ^†^: significant interaction between temperature (0°C, 20°C) and exercise (pre- and post-exercise); ^#^: significantly different from 20°C, ηp^2^: partial eta squared; box plot explanation: upper horizontal line of the box, 75^th^ centile; lower horizontal line, 25^th^ centile; horizontal bar within the box, median; upper horizontal bar outside the box, 90^th^ centile; lower bar outside the box, 10^th^ centile

**Figure 3:**
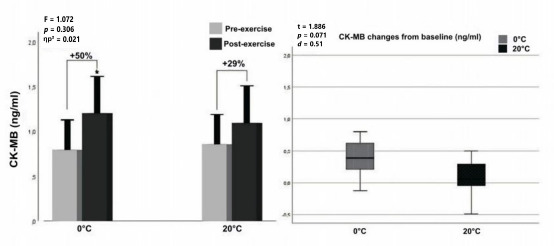
Graphical representation of CK-MB concentration pre- and post-exercises (left panel) and net change from baseline (right panel). Notes: *: significantly different from pre-exercise value; ηp^2^: partial eta squared; box plot explanation; upper horizontal line of the box, 75^th^ centile; lower horizontal line, 25^th^ centile; horizontal bar within the box; upper horizontal bar outside the box, 90^th^ centile; lower bar outside the box, 10^th^ centile

According to two-way ANOVA analyses for CK-MB levels, no significant interaction (F = 1.072, *p* = 0.306, ηp^2^ = 0.021) was found between temperature (factor 1: 0°C vs. 20°C) and exercise (factor 2: pre- vs. post-exercise). Furthermore, no significant interaction (F = 1.072, *p* = 0.306, ηp^2^ = 0.021) was found between the temperature (factor 1: 0°C vs. 20°C) and exercise (factor 2: pre- vs. post-exercise). However, a significant main effect of exercise was observed (F = 15.987, *p* < 0.001, ηp^2^ = 0.242), indicating an overall increase in CK-MB levels from pre- to post-exercise regardless of the temperature condition. There was a significant difference between pre- and post-exercise values at the 0°C environmental temperature for serum CK-MB levels (*p* < 0.001, *d* = 0.53), whereas no significant difference was found (*p* = 0.71, *d* = 0.36) at 20°C (Figure 3, left panel). Also, in Figure 3 (right panel) one may see that the CK-MB increase from baseline at 0°C was higher than at 20°C (+50% and +29%, respectively), although this difference was not statistically significant (t = 1.886, *p* = 0.071, *d* = 0.051).

There was no significant interaction according to three-way ANOVA between the time (pre- vs. post-exercise), and the environmental temperature (0°C vs. 20°C) in both sub-groups for serum Gal-3 (males vs. females: F = 0.091, *p* = 0.765, ηp^2^ = 0.004; professional athletes and physically active individuals: F = 0.862, *p* = 0.362, ηp^2^ = 0.035) and serum CK-MB levels (males vs. females; F = 1.545, *p* = 0.226, ηp^2^ = 0.060, professional athletes and physically active individuals; F = 0.088, *p* = 0.769, ηp^2^ = 0.004).

However, in the pairwise comparisons of sub-groups between pre- and post-exercise (Tables 2 and 3), Gal-3 and CK-MB levels significantly increased at 0°C in females (Gal-3: *p* = 0.005, *d* = 0.27; CK-MB: *p* < 0.001, *d* = 1.30), males (Gal-3: *p* = 0.006, *d* = 0.08; CK-MB: *p* < 0.001, *d* = 0.51), professional athletes (Gal-3: *p* = 0.019, *d* = 0.24; CK-MB: *p* = 0.001, *d* = 0.30), and physically active individuals (Gal-3: *p* = 0.002, *d* = 0.54; CK-MB: *p* < 0.001, *d* = 1.60), whereas no significant differences were found at 20°C for Gal-3 and CK-MB levels. Also, the greatest change for Gal-3 levels (+9.0%) was in physically active individuals at 0°C, while for CK-MB levels (+100%) the greatest change was observed in females at 0°C.

**Table 2:** Gal-3 values of particular subgroups pre- and post-exercise at different environmental temperatures.

Gal-3 (ng/ml) 0°C	Pre-exercise	Post-exercise	%Change	t	*p*	*d*
Professional	21.7 ± 5.3	22.9 ± 4.9	+5.5%	−2.745	0.019	0.24
Physically active	18.7 ± 2.8	20.4 ± 3.5	+9.0%	−3.893	0.002	0.54
Females	20.1 ± 5.3	21.6 ± 5.8	+7.4%	−3.551	0.005	0.27
Males	20.1 ± 3.6	21.5 ± 3.5	+6.9%	−3.201	0.006	0.08
						
Gal-3 (ng/ml) 20°C	Pre-exercise	Post-exercise	% Change	t	*p*	*d*
Professional	22.0 ± 4.0	22.5 ± 4.3	+2.2%	−1.465	0.171	0.12
Physically active	18.6 ± 3.3	18.8 ± 3.0	+1.0%	−0.300	0.769	0.06
Females	20.3 ± 2.4	20.6 ± 2.7	+1.4%	−0.666	0.520	0.12
Males	20.1 ± 4.8	20.5 ± 4.9	+1,9%	−0.671	0.513	0.08

Notes: Values in bold indicate statistically significant differences (p < 0.05)

**Table 3:** CK-MB values of particular subgroups pre- and post-exercise at different environmental temperatures.

CK-MB (ng/ml) 0°C	Pre-exercise	Post-exercise	%Change	t	*p*	*d*
Professional	0.94 ± 1.04	1.26 ± 1.12	+34%	−4.529	0.001	0.30
Physically active	0.66 ± 0.30	1.14 ± 0.38	+72%	−8.342	< 0.001	1.60
Females	0.40 ± 0.29	0.81 ± 0.44	+100%	−6.499	< 0.001	1.30
Males	1.07 ± 0.84	1.48 ± 0.89	+38%	−5.919	< 0.001	0.51
						
CK-MB (ng/ml) 20°C	Pre-exercise	Post-exercise	% Change	t	*p*	*d*
Professional	0.69 ± 0.46	0.84 ± 0.52	+21%	−1.176	0.264	0.33
Physically Active	0.65 ± 0.53	0.89 ± 0.70	+36%	−1.446	0.172	0.39
Females	0.77 ± 0.64	1.12 ± 0.76	+45%	−1.745	0.112	0.54
Males	0.60 ± 0.34	0.68 ± 0.40	+13%	−0.820	0.426	0.23

Notes: Values in bold indicate statistically significant differences (p < 0.05)

Post-exercise Gal-3 levels at 0°C showed a significant increase compared to pre-exercise levels (*p* < 0.001), with a small to moderate effect size (Cohen’s *d* = 0.33; 95% CI: 0.14–0.52). At 20°C, the increase was not significant (*p* = 0.368; *d* = 0.09; 95% CI: –0.11 to 0.29).

For CK-MB, the change at 0°C was statistically significant (*p* < 0.001; *d* = 0.53; 95% CI: 0.30–0.76), indicating a moderate effect, while no significant difference was observed at 20°C (*p* = 0.71; *d* = 0.36; 95% CI: 0.08–0.64). Although the percentage increase in CK-MB at 0°C (+50%) exceeded that at 20°C (+29%), the between-condition difference did not reach statistical significance (t = 1.886, *p* = 0.071; *d* = 0.051; 95% CI: –0.004 to 0.106).

## Discussion

CVD remains a leading global cause of mortality and morbidity, and while regular aerobic exercise is widely recognized for its protective effects, emerging evidence suggests that certain exercise conditions—particularly high intensity and extreme environmental exposures—may paradoxically induce transient myocardial stress. Cold-weather exercise, in particular, has been shown to increase sympathetic activity, peripheral vasoconstriction, and myocardial oxygen demand, all of which could heighten cardiovascular strain. However, the physiological consequences of combining aerobic exercise with cold exposure remain poorly understood, especially in healthy individuals.

Gal-3 is a biomarker that reflects early-stage cardiac fibrosis and inflammatory processes, while CK-MB serves as a conventional indicator of myocardial damage. Although both markers have been individually examined in the context of exercise, their simultaneous response to physical activity under varying temperature conditions has not been comprehensively evaluated. Investigating these markers in a cold-exercise setting may help clarify whether exposure to low temperatures augments cardiac biomarker responses in a way that signals early myocardial strain. Therefore, this study aimed to investigate the acute effects of aerobic exercise performed at 0°C and 20°C on serum levels of Gal-3 and CK-MB in healthy young adults with varying fitness levels. By comparing biomarker responses under these two environmental conditions, the study sought to determine whether cold-weather exercise would pose an additional cardiovascular burden, potentially contributing to early myocardial stress even in the absence of overt pathology.

We observed that baseline Gal-3 levels of professional athletes and physically active individuals were significantly higher than those of sedentary individuals. Post-exercise serum Gal-3 levels were significantly higher than the pre-exercise values at the 0°C environmental temperature. Post-exercise serum CK-MB levels increased significantly at the 0°C environmental temperature. According to our literature review, this was the first study to evaluate the effects of aerobic exercise performed at two different temperatures on cardiac damage, by measuring Gal-3 and CK-MB levels.

As seen in previous studies evaluating the effects of exercise in cold weather on irisin and adropin levels as well as CTRP-3 levels, exercising under cold conditions may have different effects on human physiology. Aerobic exercise in the cold leads to significantly increased CTRP3 and Irisin levels compared to exercise at a neutral temperature, but does not cause a significant increase in adropin levels (Sebin et al., 2024; Ulupinar et al., 2021).

### Exercise Type Can Affect Cardiovascular Health

Long-term regular exercise reduces the risk of the development of many diseases, such as cardiovascular diseases (Lavie et al., 2015). However, excessive exercises that markedly increase the cardiac workload can cause cardiac inflammation and fibrosis, leading to cardiac pathologies and damage (Knechtle and Nikolaidis, 2018). The relationship among exercise, damage/adaptation, and biomarker variation is complicated due to the number of variables involved, including the exercise type, intensity, duration, and training background (Pingitore et al., 2015). Eijsvogels et al. (2015) found that increases in cTns’ levels were directly correlated to the exercise duration, probably because longer competitions require higher intensity exercise. Matsuo et al. (2014) reported that a 13-min high-intensity aerobic-type interval cycling program performed five times per week was a highly effective and time-efficient method for enhancing VO_2max_, cardiac mass, and overall heart function in sedentary individuals.

### Galectin 3 in the Human Body

Gal-3 is a stable biomarker for heart failure and the risk of mortality due to other pathologies; it is not associated with age, the body mass index, or sex, and it is stable for nine freeze-thaw cycles after storage at −20°C or −70°C (Dong et al., 2018; Meijers et al., 2014). Gal-3 is mainly synthesized in the macrophages and lower amounts in cells and secreted to the extracellular matrix; thus, it is present in the circulation (Suthahar et al., 2018). Oxidative stress can be induced by increasing oxidants’ release and decreasing the antioxidants by Gal-3 (Suzuki et al., 2008). Gal-3 plays an important role in acute and chronic inflammatory responses, such as chemoattraction, opsonization, and degranulation of immune cells. It can cause cardiac fibrosis, leading to systolic and diastolic heart failure (Wright et al., 2017). Issa et al. (2015) found that Gal-3 levels in healthy controls who reached 70–80% of their maximal heart rate capacity (maximum heart rate: 220 − age) increased to maximum levels between 1 and 3 hours compared to baseline after aerobic exercise.

### Galectin Levels in Exercise

Gal-3 plays an important role in immune and inflammatory processes and is recognized as a prognostic biomarker in cardiovascular disease (Hrynchyshyn et al., 2013). Elevated Gal-3 levels are associated with increased mortality and heart failure risk, and their use for cardiac risk assessment is endorsed by major cardiology societies. Unlike NT-proBNP, which reflects pressure overload, Gal-3 provides early insight into cardiac fibrosis and remodeling. Although only few studies have explored the link between Gal-3 and exercise, intense endurance activities (e.g., marathons) have been shown to raise Gal-3 levels without corresponding increases in traditional cardiac markers such as troponins or NT-proBNP (Le Goff et al., 2019). This rise may stem from inflammation or inadequate recovery rather than pathological damage. However, whether Gal-3 elevation reflects adaptive muscle response or early cardiac injury remains uncertain. Current evidence suggests Gal-3 increases after vigorous exercise are part of the inflammatory response, but it is still unclear whether this indicates physiological adaptation or potential harm (Le Goff et al., 2020).

While Gal-3 is widely recognized for its role in cardiac fibrosis and inflammation, emerging evidence suggests that skeletal muscle may also be a source of circulating Gal-3, particularly following physical exertion. Hättasch et al. (2014) demonstrated in a dual model study involving both human athletes and mice that Gal-3 levels rose post-exercise, with skeletal muscle identified as a significant contributor. This suggests that elevations in Gal-3 after endurance activity may reflect systemic responses involving both muscle repair and immune activation, rather than cardiac stress alone. Therefore, in our study, the post-exercise increases in Gal-3—especially in the absence of structural cardiac changes—might partly originate from skeletal muscle stress or remodeling. This highlights the need for careful interpretation of Gal-3 levels in exercise settings and supports the inclusion of additional markers or imaging to differentiate cardiac from non-cardiac sources.

In this study, baseline Gal-3 values were highest in professional athletes, followed by physically active participants, and the lowest in sedentary people, similarly to a study by Le Goff et al. (2020).

Additionally, baseline Gal-3 levels were higher than the normal range (< 17.8 ng/mL) in professional athletes and the physically active group. There can be three reasons for this result. Firstly, this may be due to training several times a week and insufficient recovery to return to the normal basal rate; secondly, it may be due to the development of cardiac fibrosis because of chronic exercise or, finally, cardiac remodeling that develops due to long-term exercise and related factors. Similarly to our results, Hättasch et al. (2014) found that baseline Gal-3 levels were higher in athletes compared to the sedentary group.

Exercise is recommended to prevent cardiovascular diseases. Hättasch et al. (2014) evaluated Gal-3 levels in athletes and mice in the same study and demonstrated that baseline Gal-3 levels of athletes were normal although higher than in the sedentary and healthy control groups. While the systolic function of the left and right ventricles remained unchanged, levels of Gal-3 significantly increased after endurance exercise (30 km). High-intensity exercise has been reported to lower galectin-3 levels, a biomarker linked to heart failure risk, especially among postmenopausal women (Keyhani et al., 2020).

According to their results, Hättasch et al. (2014) attributed the increase in Gal-3 levels to exercise-induced inflammatory response, together with leukocytes and pro-inflammatory cytokines. Furthermore, no significant correlation was found between echocardiographic and cardiac magnetic resonance imaging results and cardiac function, fibrosis, and baseline Gal -3 levels. In the same study, it was determined that Gal-3 levels in the active mice group were significantly increased compared to the sedentary mice group. It was shown that most of the source of Gal-3 originated from skeletal muscle, and only a very small part from the left ventricular myocardium (Hättasch et al., 2014).

The relationship between endurance exercise and Gal-3 levels remains subject to debate. Hättasch et al. (2014) reported increased Gal-3 levels in endurance athletes following running, despite the absence of myocardial or functional cardiac alterations. Similarly, Salvagno et al. (2014) observed significant elevations in Gal-3, high-sensitivity troponin I (hs-TnI), and NT-proBNP levels after a 60-km ultramarathon, suggesting potential subclinical myocardial stress. Le Goff et al. (2020) found that Gal-3 levels rose immediately after marathon (42 km), ultra-trail (67 km), and recreational runs, with the most pronounced increase observed in marathon participants, followed by a rapid decline within three hours. These findings imply that Gal-3 responses may be more closely related to exercise intensity than duration. In contrast, baseline Gal-3 levels in sedentary individuals remained within the normal range, likely reflecting their younger age and absence of underlying cardiac pathology. Collectively, these studies suggest that prolonged or high-intensity endurance exercise may induce transient elevations in Gal-3 and other cardiac biomarkers, potentially reflecting early or reversible cardiac stress.

Vassalle et al. (2018) found that Gal-3, pro-BNP, and cardiac TnT levels were transiently increased after a half-marathon (21 km). Elevation of these markers could be a part of an adaptive mechanism to exercise with long-term endurance training, which served as a stimulative stress factor for physiological adaptation, and it may indicate potentially harmful effects of exercise on the cardiovascular system. In the same study, cTnI and AAT did not change significantly.

A short-term increase in Gal-3 levels does not cause cardiac problems. For stimulating fibrocytes and developing cardiac fibrosis, longer exposure to high levels of Gal-3 is required. Although it is a stable biomarker, the time for normalization of post-exercise levels varies among individuals. The cause of these differences and their effects on the heart are unknown; thus, there is a need for extensive studies on this subject (Le Goff et al., 2019).

### CK-MB Levels in Exercise

In a study conducted with 11 healthy young male participants, it was determined that hs-cTnT levels increased significantly compared to baseline immediately after and 1 hour after 40 minutes of aerobic exercise in intermittent and continuous forms. It was determined that continuous exercise contributed more to the hs-cTnT increase than intense exercise. Furthermore, there was no significant change in CK-MB levels (Ranjbar et al., 2017). Cardiac Tn levels increase moderately both during and following physical exercise, which represents a physiological phenomenon. Previous echocardiographic research suggested a relationship between cardiac Tn levels and cardiac dysfunction following exercise (Aengevaeren et al., 2019). However, a study using cardiac magnetic resonance imaging (MRI) did not find any evidence of cardiac damage or fibrosis (Stavroulakis and George, 2020).

To the best of our knowledge, none of the previous studies have examined the effects of aerobic exercise under cold weather conditions on cardiovascular fibrosis or damage. Valtonen et al. (2018) found that submaximal exercise and cold weather had an additive effect on the increment of the cardiac workload by increasing systolic blood pressure and the heart rate in stable CAD patients. Those results showed that aerobic exercise in a cold environment might increase the cardiac workload and stress in athletes. As is known, insufficient coronary circulation in CAD patients can cause myocardial ischemia and injury during exercise in cold weather via discordance between the exigency of oxygen and procurement. Thus our study is unique in a sense that it investigated the effects of aerobic exercise combined with cold weather compared with neutral weather in healthy athletes.

While post-exercise increases in Gal-3 and CK-MB—particularly under cold conditions—were statistically significant, their clinical relevance in healthy individuals remains uncertain. The magnitude of change in Gal-3 (e.g., +7.1% at 0°C) and CK-MB (+50% at 0°C) levels did not exceed established thresholds associated with pathological cardiac injury or dysfunction. These elevations are more likely indicative of transient physiological stress rather than myocardial damage, especially in the absence of symptoms or structural cardiac changes. Therefore, while these findings suggest heightened cardiac biomarker sensitivity to cold-exercise stress, they should be interpreted as subclinical and not necessarily predictive of adverse outcomes in this population.

The observed elevations in Gal-3 and CK-MB levels following aerobic exercise at 0°C suggest that cold exposure may amplify cardiac stress responses, even in healthy and well-trained individuals. For athletes who regularly train or compete in cold environments, these findings highlight the importance of closely monitoring training intensity, duration, and recovery, especially during early-season conditioning or cold-weather endurance events. Although the biomarker changes in our study appear to reflect physiological, rather than pathological, stress, repeated or cumulative exposure to such conditions may have long-term implications for myocardial adaptation or remodeling. Incorporating adequate warm-up routines, layered thermal protection, and recovery protocols may help mitigate cold-induced cardiovascular strain. Future research should investigate whether these transient biomarker shifts correlate with functional cardiac changes over time, particularly in endurance athletes or individuals with predisposing cardiac risk factors.

## Limitations

The present study has several limitations. Firstly, this was a single-center study. This offers advantages concerning the standardization of the determination of Gal-3 and CK-MB serum levels. The effects of aerobic exercise in the cold on myocardial damage by increasing the workload of the heart and decreasing its oxygenation can be due to the decrease in partial oxygen pressure at high altitudes. Since 40 minutes of aerobic exercise could not be performed in the sedentary group, post-exercise Gal-3 levels could not be evaluated and compared with others. We performed a single Gal-3 and CK-MB measurement, and it remains unclear in which manner these biomarkers vary in time. In addition, for ethical reasons, during the exercise at 0°C, athletes were protected from the cold with three-layer clothing, thus contact with cold air was limited to the face and hands only. Additionally, ECG/Echo assessments were not included, as prior research suggested that exercise in cold environments did not always lead to detectable changes. At the same time, biochemical markers (Gal-3 and CK-MB) are more responsive indicators of myocardial stress. Only pre- and post-exercise biomarker measurements were taken, so time-dependent variations remain unclear. The modest sample size may limit broader applicability. The lack of exercise in the sedentary group also limits comparative power.

Moreover, the absence of cardiac imaging tools such as echocardiography or cardiac MRI limits our capacity to evaluate functional or structural changes in the heart directly. As a result, although the observed shifts in serum biomarkers may point to temporary cardiac stress, we cannot conclusively determine whether subclinical myocardial injury or remodeling has occurred. Additionally, because biomarker measurements were taken only before and immediately after exercise, we were unable to capture their progression over time—such as peak values or recovery trends—making it difficult to fully interpret the timing and clinical significance of these changes.

## Conclusions

Baseline Gal-3 levels of professional athletes and physically active individuals were significantly higher than those of sedentary individuals. Gal-3 levels increased dramatically after 40 minutes of aerobic exercise at the 0°C environmental temperature. Additionally, there was a significant difference between pre- and post-exercise values at the 0°C environmental temperature for serum CK-MB levels (*p* < 0.001), while there was no significant difference (*p* = 0.71) at 20°C.

These findings suggest that acute aerobic exercise under cold conditions may provoke measurable changes in cardiac-related biomarkers, particularly Gal-3 and CK-MB. However, without complementary assessments such as cardiac imaging or functional testing, the clinical implications of these biochemical elevations should be interpreted with caution. While the observed changes may indicate transient physiological stress responses rather than definitive myocardial damage or fibrosis, the results highlight the potential importance of environmental conditions on cardiovascular biomarker profiles. Future investigations should explore the prolonged impact of exercising in cold environments on heart health, ideally by integrating advanced imaging techniques like cardiac MRI or echocardiography to establish links between biomarker fluctuations and structural heart changes. Broader biomarker panels—including high-sensitivity troponins, NT-proBNP, and ST2—may provide a more in-depth view of cardiac stress responses. Studies tracking Gal-3 and CK-MB levels at multiple time points post-exercise could shed light on their dynamic profiles. Research should also assess how varying exercise types and intensities, as well as factors such as age, sex, and environmental conditions, influence outcomes. Finally, involving individuals with heart disease in future studies could help refine exercise recommendations for at-risk populations.
